# Compressive peroneal neuropathy by an intraneural ganglion cyst combined with L5 radiculopathy: A case report

**DOI:** 10.1097/MD.0000000000017865

**Published:** 2019-11-01

**Authors:** Se-Heum Park, Hwan-Kwon Do, Geun-Yeol Jo

**Affiliations:** Department of Physical Medicine and Rehabilitation, Haeundae Paik Hospital, Inje University College of Medicine, Busan, Republic of Korea.

**Keywords:** common peroneal nerve, electrodiagnostic examination, foot drop, intraneural ganglion cyst, L5 radiculopathy

## Abstract

**Rationale:**

Most cases of foot drop are known to result from lower motor neuron pathologies, particularly lumbar radiculopathy and peripheral neuropathy, including common peroneal neuropathy. To improve the prognosis of foot drop, it is important to quickly and accurately diagnose the etiology and provide appropriate treatment.

**Patient concerns:**

A 65-year-old female patient with a history of L4-5 intervertebral disc herniation presented with right foot drop that had developed 1 month previously.

**Diagnosis:**

Electrodiagnostic examination revealed common peroneal neuropathy combined with L5 radiculopathy, with the former being the main cause of the foot drop. MRI of the right knee was performed to identify the cause of the peroneal nerve lesion, which revealed an intraneural ganglion cyst in the common peroneal nerve.

**Interventions:**

The patient was treated by ultrasound-guided percutaneous cyst aspiration and corticosteroid injection into the decompressed ganglion, followed by strengthening exercise, electrical stimulation therapy, and prescription of an ankle foot orthosis.

**Outcomes:**

We confirmed regeneration of the injured peroneal nerve at the follow-up electrodiagnostic examination 12 weeks after the intervention. In addition, the manual motor power test demonstrated an increase in the ankle dorsiflexor function score by one grade.

**Lessons:**

Diagnosing the cause of foot drop can be difficult with multiple co-existing pathologies, and consideration of various possible etiologies is the key for appropriate diagnosis and treatment. In addition to imaging modalities such as MRI, electrodiagnostic examination can help to improve diagnostic accuracy. Intraneural ganglion cyst of the common peroneal nerve is rare, but should be considered as a possible cause of foot drop.

## 1 Introduction

Foot drop is a medical term typically referring to weakness of the dorsiflexor muscles of the foot. Lower motor neuron causes of foot drop include common peroneal mononeuropathy, sciatic mononeuropathy, lumbosacral plexopathy, and severe L5 radiculopathy. There are also upper motor neuron causes, such as spinal cord lesion and parasagittal frontal lobe lesion^[1]^; however, the majority of foot drop cases result from lower motor neuron pathology, which can be broadly divided into lumbar radiculopathy, particularly L5 radiculopathy, and peripheral neuropathy. The most important peripheral neuropathy is the common peroneal mononeuropathy, which is the most common entrapment neuropathy of the lower extremity.^[1–4]^

Accurately differentiating the peripheral etiology of foot drop is difficult because the main dorsiflexor muscle of the ankle, tibialis anterior (TA), is supplied by the peroneal nerve that receives a significant contribution from the L5 nerve root. Preoperative muscle strength and palsy duration are statistically significant predictors of foot drop improvement. Therefore, to identify the etiology of foot drop and achieve prompt, accurate diagnosis and treatment, history taking, physical examination, and selection of appropriate diagnostic tools are important.^[4]^

Herein, we report our experience of the diagnosis and treatment of foot drop caused by an intraneural ganglion cyst of the common peroneal nerve in combination with L5 radiculopathy due to lumbar disk herniation.

## 2 Case presentation

**Figure 1:**
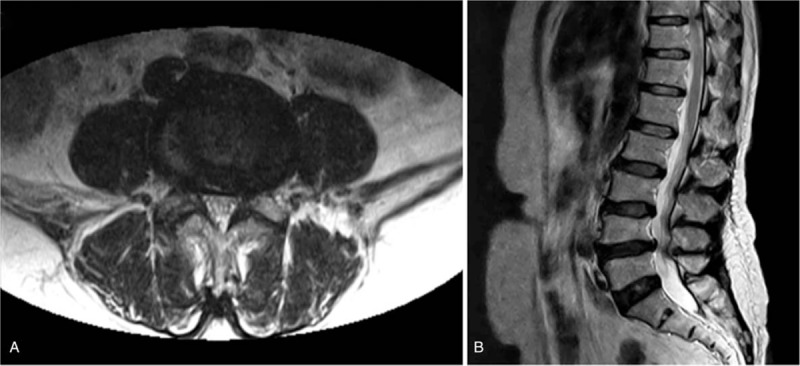
T2-weighted magnetic resonance images of the lumbar spine. (A) Axial image at the lumbar 4-5 level; (B) coronal image of the lumbar spine.

A 65-year-old female presented with right foot drop that first developed 1 month earlier. The patient was a homemaker and had no history of other diseases. She denied any recent trauma or excessive exercise. Two months previously, the patient had developed back pain with radiating pain to the lateral side of her right lower leg. Lumbar spine magnetic resonance imaging (MRI) revealed herniation of the L4–5 intervertebral disc and conservative treatment was prescribed, including medication for pain control (Fig. 1). Even though the patient's pain improved, she developed right foot drop one month later and was referred to our hospital for further evaluation and treatment.

On neurologic examination, the patient demonstrated steppage gait. She had numbness and a tingling sensation along the distribution of the L5 dermatome at the right lower leg, including the lateral lower leg and foot dorsum, and there was slight atrophy of the right lower leg anterior and lateral compartments. The straight leg raising test was negative and deep tendon reflex testing demonstrated that both knee jerk and ankle jerk reflexes were normal. Manual muscle testing (MMT) revealed a grade 1 (trace) right ankle dorsiflexion as well as ankle eversion, and grade 5 (normal) right ankle plantarflexion and knee extension.

**Figure 2:**
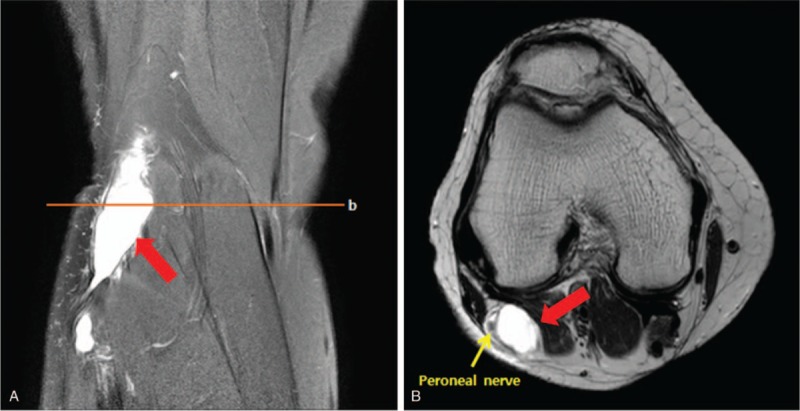
T2-weighted magnetic resonance images of the knee. (A) Coronal image of the popliteal fossa; (B) axial image at the distal femur level. The red arrows indicate the intraneural ganglion cyst in the common peroneal nerve. The yellow arrow indicates the compressed peroneal nerve.

We performed electrodiagnostic examination to precisely identify the compromised nerve lesion using Viking Select (Nicolet, San Carlos, CA). The motor nerve conduction study showedcomplete conduction block in the right peroneal nerve at the fibula head level, whereas the sensory nerve conduction study showed no sensory nerve action potentials in the right superficial peroneal nerve. The test results for the right tibial and sural nerves were within normal limits (Table 1). In needle electromyography, we observed denervation potentials (200–300 μV) and substantially reduced volitional motor unit action potentials (MUAPs) in the right TA, peroneus longus (PL), and extensor hallucis longus (EHL) muscles. In addition, we observed denervation potentials (<50 μV) in the right tibialis posterior (TP) and L5-level paraspinal muscles, whereas volitional MUAPs were in the normal range (Table 2). According to the electrodiagnostic findings, the right common peroneal nerve was the main cause of the foot drop and was accompanied by L5 radiculopathy. Therefore, knee MRI was performed to identify the cause of the peroneal nerve lesion. A T2 hyperintense lesion was found in the peroneal nerve at the proximal tibia level with a maximum diameter of 2.0 × 1.5 × 3.8 cm (Fig. 2). Based on these findings, a diagnosis of common peroneal intraneural ganglion cyst was made.

**Table 1:**
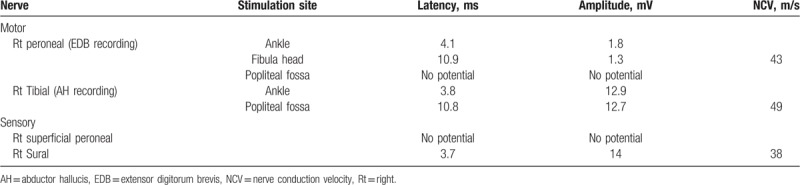
Results of nerve conduction study.

**Table 2:**
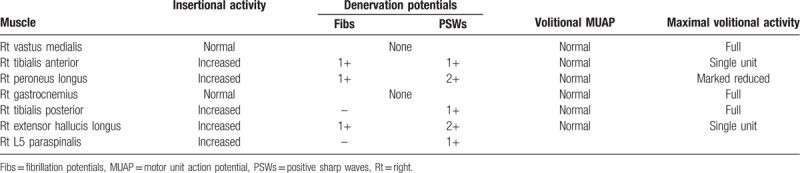
Results of needle electromyography.

**Figure 3:**
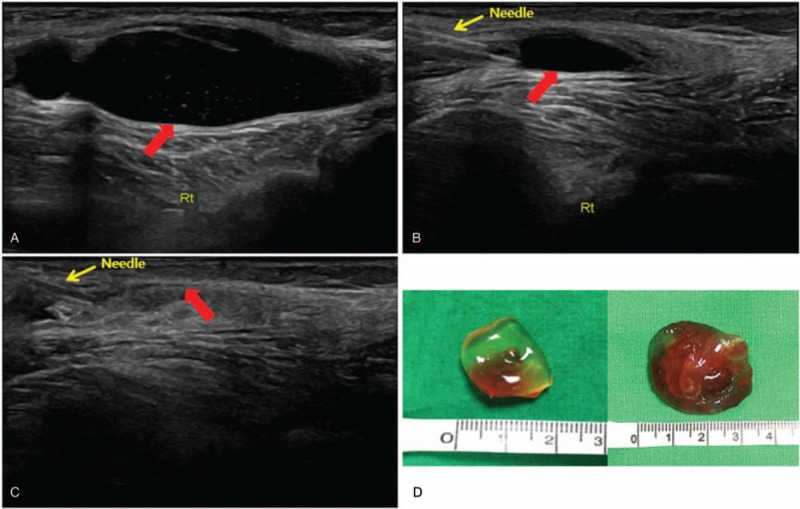
Longitudinal ultrasound images before and after cyst aspiration. (A) View of the intraneural ganglion cyst in the lateral popliteal fossa. (B) View during cyst aspiration. (C) View of the shrunk cyst after aspiration. (D) Two aspirated gelatinous thick mucoid specimens. The red arrows indicate the intraneural ganglion cyst of the common peroneal nerve. The yellow arrows indicate the inserted needle.

The patient declined surgical treatment for the cyst due to the risk of surgical complications. Instead, ultrasound-guided percutaneous cyst aspiration was performed using a 16-gauge needle, followed by corticosteroid (triamcinolone) injection. We aspirated 8 cc of yellow-colored, thick, gelatinous, mucoid content from the cyst. The shrunk cyst was confirmed by ultrasonography after aspiration (Fig. 3). The patient was asked to perform dorsiflexor strengthening exercises thereafter using an elastic band. Electrical stimulation therapy was applied to the TA and PL muscles to prevent atrophy. An ankle foot orthosis (AFO) was prescribed to correct the steppage gait and prevent toe dragging while walking.

Twelve weeks after aspiration, there were improvements in ankle dorsiflexion and big toe extension to MMT grade 2. The tingling sensation on the lateral side of the right lower leg subsided, but the numbness still remained. A follow-up electrodiagnostic examination showed some recovery of the conduction block in the right peroneal nerve and sensory nerve action potentials were newly detected in the superficial peroneal nerve. Furthermore, needle electromyography showed polyphasic MUAPs in the TA, PL, and EHL muscles, indicating regeneration of the peroneal nerve (Table 3). To date, there are no findings to indicate cyst recurrence. The patient still uses an AFO, performs self-exercise using elastic bands, and undergoes electrical stimulation therapy.

**Table 3:**
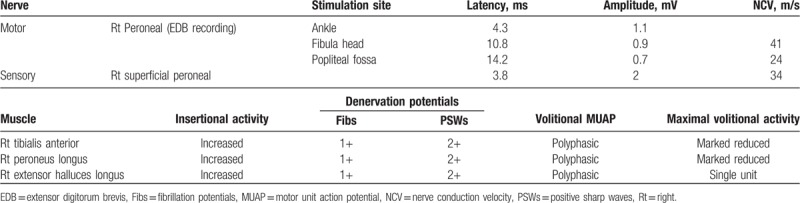
Follow-up electrodiagnostic studies.

## 3 Discussion

Foot drop is a common clinical symptom, the most common cause of which is known to be peroneal neuropathy. However, a cautious diagnostic approach is required as there are numerous other possible causes. A history of surgery or trauma can help differentiate the causes, and during physical examination, it is important to carefully examine the function of not only the ankle dorsiflexors, but also the TP, gastrocnemius, the hamstrings, and the gluteal muscles. This is because these muscles are affected by proximal nerve lesions, such as those of the L5 nerve root, lumbosacral plexus, and sciatic nerve, which have similar presentations as peroneal nerve lesions.^[2]^ Thus, thorough history-taking and physical examination should be performed first. Next, if the exact cause of foot drop is difficult to identify, or if any cause other than peroneal neuropathy is suspected, electrodiagnostic examinations, including nerve conduction test and electromyography, are helpful for localization of the lesion, and can also help predict the prognosis or follow-up course of recovery. In patients diagnosed with peroneal neuropathy, if the etiology is unclear or the symptoms become worse, imaging of the knee is required.^[2]^ Compared to other imaging modalities, including ultrasonography and computed tomography, knee MRI is known to demonstrate excellent soft tissue contrast and multiplanar images for identifying nerve lesions.^[5]^

In this case, the patient was diagnosed with L5 radiculopathy and common peroneal neuropathy, both of which may cause foot drop. In the needle electromyography, the amplitude of the denervation potential was much lower in the TP and L5-level paraspinal muscles than that in other denervated muscles and the volitional MUAPs in the TP were fully preserved. In the nerve conduction study, both the motor and the sensory conduction in the right peroneal nerve were absent. On the knee MRI, the peroneal nerve was displaced to the posterolateral side of the cyst by the intraneural ganglion cyst and severe compression was confirmed (Fig. 2B). Considering the patient's history and all diagnostic findings, she was diagnosed with right peroneal neuropathy combined with L5 radiculopathy, and the peroneal neuropathy caused by the intraneural ganglion cyst was determined to be the main cause of the patient's foot drop. Furthermore, the improvement in foot drop after peroneal nerve decompression supported our judgement that it was caused by peroneal neuropathy due to the intraneural ganglion cyst.

An intraneural ganglion cyst is a nonneoplastic mucinous cyst that is formed by the accumulation of thick mucinous fluid in the epineurium of a peripheral nerve.^[6]^ The cyst can compress the adjacent nerve fascicle, causing symptoms such as local or radiating pain, paresthesia, weakness, muscle denervation, and atrophy.^[6–9]^ In the lower extremity, the peroneal nerve is known to be most commonly involved at the knee level and at the level of the proximal tibiofibular joint.^[8,10–12]^ Intraneural ganglion cysts are most commonly reported in middle-aged males with a previous history of trauma and are accompanied by neurologic symptoms, including foot drop and lower extremity paresthesia. As the etiology of intraneural ganglion cysts is controversial, treatments are diverse and still debated.^[6,11]^ Furthermore, the recurrence rate is reported to be high following treatment and is estimated to be 11% to 30% after surgical treatment.^[13,14]^ Although surgical treatment is invasive and associated with complications, such as permanent nerve damage, vessel injury, and infection, it is currently considered to be the standard treatment procedure. One possible minimally invasive alternative to surgical treatment is ultrasound-guided percutaneous cyst aspiration and corticosteroid injection into the decompressed ganglion.^[14]^

When multiple possible causes of foot drop are combined, it can be difficult to decide on the most appropriate management. As our case demonstrates, it is important to differentiate between spinal nerve root lesions and peripheral nerve lesions to provide appropriate early treatment, as early treatment is a significant predictor of foot drop improvement.^[4]^ In addition, following the appropriate treatment, the patient's prognosis can be improved by individual rehabilitative approaches, including continued exercise and electrical stimulation therapy to prevent denervated muscle atrophy, as well as AFO use to reduce fall risk.^[15]^ Continual follow-up to monitor for recurrence of the ganglion cyst is also recommended.

## 4 Conclusion

Foot drop has various etiologies, making it important to identify the correct cause for diagnosis and treatment. Intraneural ganglion cyst of the peroneal nerve is rare, but should be considered as a potential cause of foot drop. The differential diagnosis can be difficult based solely on physical examination and radiologic tests. Therefore, to obtain an accurate diagnosis, it is crucial to consider the various different causes of foot drop and electrodiagnostic examinations help to improve diagnostic accuracy.

## Author contributions

**Conceptualization:** Hwan-Kwon Do.

**Resources:** Se-Heum Park, Hwan-Kwon Do, Geun-Yeol Jo.

**Supervision:** Geun-Yeol Jo.

**Writing – original draft:** Se-Heum Park.

**Writing – review & editing:** Hwan-Kwon Do.

Hwan-Kwon Do orcid: 0000-0002-5862-4233.
